# The Administrative Side of Sanatorium Work

**Published:** 1909-07-10

**Authors:** 


### Nursing Administration.

### THE ADMINISTRATIVE SIDE OF SANATORIUM WORK.

### I. NURSING.

The last few years have seen an enormous extension of sanatorium work, and there seems reason to believe that this branch of curative treatment is only at the commencement of its development. The open-air colony for working patients is likely to become, as time goes on, an indispensable part of municipal sanitary effort, and its success will depend in great measure on the degree to which it can be made self-supporting. Since it has been discovered that it is not merely unnecessary, but actually harmful, to keep the consumptive in idleness, numerous more or less paying industries have grown up in connection with open-air treatment. But one and all demand a trained method of treatment, and the maintenance of a strict rule of life, through which residence in the sanatorium approximates to invalid life, even although in many particulars it diverges widely from it.

In that most essential part of modern treatment for the sick the nursing, sanatorium life differs radically both from that of the hospital and from that of the convalescent home. In an ordinary convalescent home the patient is hardly considered to require nursing at all, but merely rather more attention to health than is incumbent on people in sound health. In the sanatorium he undergoes a very exact form of treatment, but instead of surrendering himself blindly, as in hospital, to the dictates and care of the nurse in attendance, he is required to learn the art of nursing himself. Every action of his day, almost every movement he makes, is thought out beforehand, and is done in conformity to a rule which he is to have securely printed on his mind. Hence the task before the managers of a sanatorium is not so much to nurse their patients as to train them to nurse themselves and one another.

The keystone of the sanatorium, as of the hospital household, is the matron. There is danger that the admitted attractiveness of this work, the opportunities it offers to a woman of resource and ability to reveal her powers for the good of others, may induce managers to whittle down the salaries of these posts to the lowest point which will enable an educated woman to provide for personal necessities. It is perfectly true that work of this description, like the work of the physician and the artist, is not to be measured by a money standard. But it is also true that only by offering an adequate salary can the managers deserve to retain and secure the services of capable administrators, thoroughly qualified to inspire and direct the difficult work carried on in the sanatorium. The matron should be a fully-trained nurse from a first-rate training school, perfectly familiar with the routine through which raw beginners are transformed into responsible heads of departments, and accustomed to make the best of indifferent material. She should have passed some time in one of the modern open-air sanatoriums where the patients are exercised in graduated work, and she should be familiar with every phase of pulmonary disease. She should have a good working knowledge of accounts, and must understand practical housekeeping. She must have the instincts of a good colonial, with the power of conforming to unfamiliar circumstances, where life is somewhat of a perpetual picnic, and the ordinary formalities which the housekeeper loves must be dispensed with altogether. A conventional matron, always striving to introduce institution routine into outdoor life, will not only be miserable herself, she will reduce all her subordinates to despair.

In order not merely to keep down expenses but to maintain the feeling of self-help, the nursing must be kept down to the lowest possible level. Bad cases, unlikely to respond to treatment, will not be received. Should patients develop serious symptoms, it is wiser to remove them immediately to other institutions. Therefore, under normal conditions, nursing duties are light. The patients are trained from the beginning to take their own temperatures. The record of their pulse falls to the nurses, and newcomers who are confined to bed must be attended to. There is no night work, and the matron will probably think little of occasionally rising to visit a patient herself whose symptoms give rise to anxiety. Under normal circumstances the matron will find that a staff of two nurses will suffice her for the ordinary small-sized sanatorium, containing from 20 to 40 beds. The senior nurse should have had previous experience in a sanatorium or special hospital for diseases of the respiratory organs. The other may be a probationer preparing either for sanatorium work or for entering hospital; it is found that this type of experience is frequently of the greatest service in settling the health of girls who want to be nurses, and preparing them for their future career. It is the experience of all matrons that sanatorium life is unsuited for the fully trained nurse. There may be months when she has no opportunity for doing any regular nursing, and she can hardly fail to become restless in the consciousness that her powers are becoming rusty.

Working, however, on this low staff, the matron must undoubtedly have full liberty to engage extra nurses whenever it may become necessary. With a large party of phthisical patients under her care she may at any moment find herself confronted with sudden cases of severe illness, in which removal is impossible, and the most skilled nursing is needed. She ought to be in touch with a supply of nurses on which she can draw for such emergencies, and there should be no delay such as must throw the ordinary work of the sanatorium out of gear, and strain the powers of the regular staff. The disposition to struggle on without extra help until all concerned are worn out, is not to be encouraged. Ready access to extra nurses and prompt recourse to their aid in emergency must be considered indispensable to maintaining efficiency with the lowest possible permanent staff.

